# Diaphragm-Free Fiber-Optic Fabry-Perot Interferometric Gas Pressure Sensor for High Temperature Application

**DOI:** 10.3390/s18041011

**Published:** 2018-03-28

**Authors:** Hao Liang, Pinggang Jia, Jia Liu, Guocheng Fang, Zhe Li, Yingping Hong, Ting Liang, Jijun Xiong

**Affiliations:** Science and Technology on Electronic Test and Measurement Laboratory, North University of China, Taiyuan 030051, China; lianghao_nuc@163.com (H.L.); liujianuc@163.com (J.L.); 18734915430@163.com (G.F.); lizhenuc@163.com (Z.L.); hongyingping_2014@163.com (Y.H.); liangtingnuc@163.com (T.L.); xiongjijun@nuc.edu.cn (J.X.)

**Keywords:** fiber-optic, Fabry-Perot, FBG, gas pressure sensor, high temperature

## Abstract

A diaphragm-free fiber-optic Fabry-Perot (FP) interferometric gas pressure sensor is designed and experimentally verified in this paper. The FP cavity was fabricated by inserting a well-cut fiber Bragg grating (FBG) and hollow silica tube (HST) from both sides into a silica casing. The FP cavity length between the ends of the SMF and HST changes with the gas density. Using temperature decoupling method to improve the accuracy of the pressure sensor in high temperature environments. An experimental system for measuring the pressure under different temperatures was established to verify the performance of the sensor. The pressure sensitivity of the FP gas pressure sensor is 4.28 nm/MPa with a high linear pressure response over the range of 0.1–0.7 MPa, and the temperature sensitivity is 14.8 pm/°C under the range of 20–800 °C. The sensor has less than 1.5% non-linearity at different temperatures by using temperature decoupling method. The simple fabrication and low-cost will help sensor to maintain the excellent features required by pressure measurement in high temperature applications.

## 1. Introduction

Compared with conventional pressure sensors, fiber-optic pressure sensors are widely used for pressure measurement of gases under high temperature because of their advantages of resistance to harsh environments, immunity to electromagnetic interference, small size and high sensitivity [1,2,3,4]. Fiber optic pressure sensors are mainly based on the Mach-Zehnder interferometer, Michelson interferometer, and FP interferometer techniques [5,6,7]. Compared to other types, the FP interferometric pressure sensor, with its advantages of high sensitivity, small size and compactness, had been widely used for measuring pressure [8]. Researchers have paid much attention to the diaphragm-based fiber-optic Fabry-Perot pressure sensor. Melissinaki et al. [9] proposed a Fabry-Perot micro-optical sensing resonator fabricated by direct laser writing on the end face of a standard telecom fiber that was demonstrated for tracing the vapors of common organic solvents. Poeggel et al. [10] reported an inexpensive all-silica fiber-optic extrinsic Fabry-Perot interferometric pressure sensor with a fiber Bragg grating added in proximity of the sensing tip, which could compensate temperature variations with 0.5 °C accuracy. Wang et al. [11] presented a fiber Fabry-Perot interferometer and a fiber Bragg grating-based pressure and temperature multiplexed sensor system with pressure and temperature measurement accuracy of 0.03 Mpa and 0.5 °C in the temperature variation range between 18 °C and 300 °C. Duraibabu et al. [12] proposed a novel miniature pressure sensor combined with a FBG for the ocean environment with a depth accuracy of about 0.01–0.03 m and resolution of ~0.5 cm. However, considering that diaphragm-based pressure sensors will suffer plastic deformation under high temperature, diaphragm-free sensors can work at higher temperatures and have a wide pressure measurement range. For instance, Villatoro et al. [13] reported an in-reflection photonic crystal fiber interferometer which exhibited high sensitivity to different volatile organic compounds. Ran et al. [14] demonstrated a diaphragm-free pressure sensor which realized pressure measurements from room temperature to 700 °C made by splicing a SMF to a photonic crystal fiber (PCF) with a hole fabricated by a 157-nm laser at the end of the PCF. Ferreira et al. [15] proposed an FP interferometric sensor with a sensitivity of 0.82 nm/MPa for pressure measurement manufactured by splicing a part of HCR-PCF to a standard SMF. Xu et al. [16] fabricated a micro-cavity fiber Fabry-Perot interferometer with a high gas sensitivity of 4.15 nm/MPa by splicing a tiny segment of a main-capillary with a feeding-capillary on one end and a SMF on the other. The measurement results of the above sensors are easily affected by the thermal expansion and the elastic modulus change of the material when applied in a high temperature environment which seriously affects the sensor accuracy. Moreover, these sensors generally have the common disadvantages of a complicated manufacturing process, high cost and the fact their working temperature is not high enough, etc.

This paper proposes a diaphragm-free fiber-optic Fabry-Perot (FP) interferometric gas pressure sensor. According to the principle whereby the gas density varies with the pressure change, pressure can be measured from room temperature to 800 °C. The sensor material is silica which has a very low temperature drift. In addition, a temperature compensation structure is designed in the sensor to offset the effect of the thermal expansion between the structures on the pressure measurement of the sensor, which further reduces the temperature drift of the sensor. The FBG which is inscribed by a femtosecond laser [17] is used for temperature decoupling. A temperature decoupling method was proposed to improve the accuracy of the sensor in high temperature applications. The sensor proposed in this paper would not suffer the problem of the plastic deformation which provides a method of pressure measurement at high temperature. The sensor has the advantages of simple structure, low cost, simple fabrication, low temperature coefficient, high accuracy and it can work stably at a high temperature of 800 °C and moreover, hopefully be used at a higher temperature.

## 2. Operating Principle

**Figure 1:**
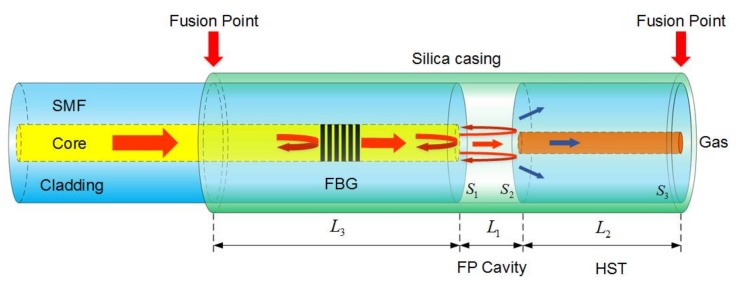
Schematic of the fiber-optic FP gas pressure sensor for high temperature.

The structure of the fiber-optic FP gas pressure sensor is shown in Figure 1. The sensor is composed of a SMF with FBG and a HST which are integrated into a silica casing from both ends. The SMF and HST is fixed and connected by silica casing and the FP cavity is formed between the ends of the SMF (S_1_) and the HST (S_2_), as shown in Figure 1. The hole of the HST provides easy access of the outside gas.

### 2.1. Optical Interference Principle

When the incident light travels along the SMF into the sensor, the light is partially reflected by the FBG, S_1_, S_2_ and the outer surface of the HST (S_3_), as shown in Figure 1. The incident light with intensity $I_{in}$ initially reflected at FBG, then the transmitted light is reflected at the two reflecting surfaces S_1_, S_2_ of the FP and S_3_, the three reflected lights interfere with each other and return to the FBG. However, the light reflected by S_3_ forms a small period fringe with a greater contrast which has an impact on the interference spectrum. Therefore, in order to get a better interference spectrum, etching S_3_ with HF acid, the light reflected at S_3_ cannot enter into the SMF which can be neglected. So the light returned to the FBG is the interference light of the FP. The intensity of the output light $I_{out}$ can be defined as [18]:

(1) $$I_{out}=I_{in}\cdot \left[f_{FBG}+{\left(1-f_{FBG}\right)}^{2}\cdot f_{FP}\right],$$

where $f_{FBG}$ and $f_{FP}$ are the reflection coefficients of FBG and FP cavity. They are defined as [18]:

(2) $$f_{FBG}=R_{1}\cdot \exp\left[-{\left(\lambda -\lambda_{FBG}\right)}^{2}/w^{2}\right],$$

(3) $$f_{FP}=2R_{2}\cdot \left[1+\cos\left(4\pi L/\lambda +\pi \right)\right],$$

where *R*_1_ is the FBG peak reflectivity, $\lambda_{FBG}$ is the FBG center wavelength, *w* is the bandwidth of the FBG reflection peak, *R*_2_ is the reflectivity of the fiber end, and *L* is the length of the FP cavity:

(4) $$L=\frac{\lambda_{1}\cdot \lambda_{2}}{2\left(\lambda_{2}-\lambda_{1}\right)},$$

where $\lambda_{1}$, $\lambda_{2}$ are the wavelength of adjacent peak-peak (or valley-valley).

**Figure 2:**
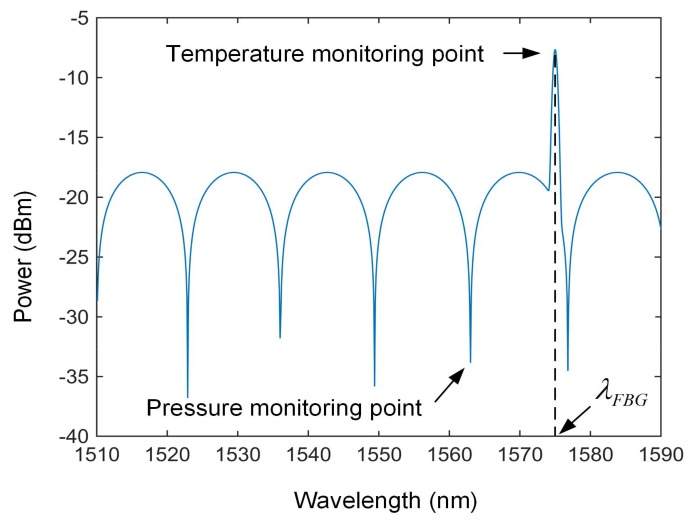
Interference spectrum simulation of the fiber-optic FP gas pressure sensor.

An interference spectrum simulation of the proposed sensor is shown in Figure 2. In the simulation, the FBG center wavelength is $\lambda_{FBG}$ = 1575 nm with peak reflectivity of 65%, the bandwidth of the FBG reflection peak is set as w = 0.5 nm, and the length of FP cavity is set as L = 90 μm. The incident light has spectrum range of 1510–1590 nm. From Figure 2, it can be seen that the temperature monitoring point corresponding to the center wavelength of the FBG ($\lambda_{FBG}$), according to the temperature sensing principle of FBG, the temperature can be calculated by the center wavelength shift of the FBG and finally achieve the purpose of temperature decoupling.

### 2.2. Temperature Decoupling Principle

At present, pressure sensors have common problems of material thermal expansion and the change of elastic modulus in high temperature environments, which directly affect the temperature drift and sensitivity of the pressure sensor. This obviously limits the applications of these pressure sensors.

In order to eliminate the effect of thermal expansion between the different materials under high temperature, a temperature compensation structure is designed in this paper. The distance between S_1_ and the left fusion point is L_3_, and the distance between S_1_ and S_2_ is L_1_, as shown in Figure 1. In our previous research [19], the experimental results showed that by setting the ratio of L_3_ and L_1_ as 9:1, the effects on the sensor due to the different coefficients of thermal expansion between the SMF and the silica casing can be eliminated. The temperature compensation structure greatly reduces the zero drift in high temperature environments.

For the problem of the change of elastic modulus under high temperature, a temperature decoupling method is presented in this paper. Initially, fitting the FBG center wavelength at different temperatures, the temperature of the sensor can be calculated by the center FBG wavelength. Next, the sensor is calibrated at a specific temperature, fitting the sensitivity to get the relationship between the temperature and sensitivity. Finally, the gas pressure can be calculated by the correspondence of the FBG center wavelength, sensitivity and temperature. Through the temperature decoupling method, the accuracy of the fiber-optic FP gas pressure sensor can be significantly improved in high temperature environments.

## 3. Sensor Fabrication

**Figure 3:**
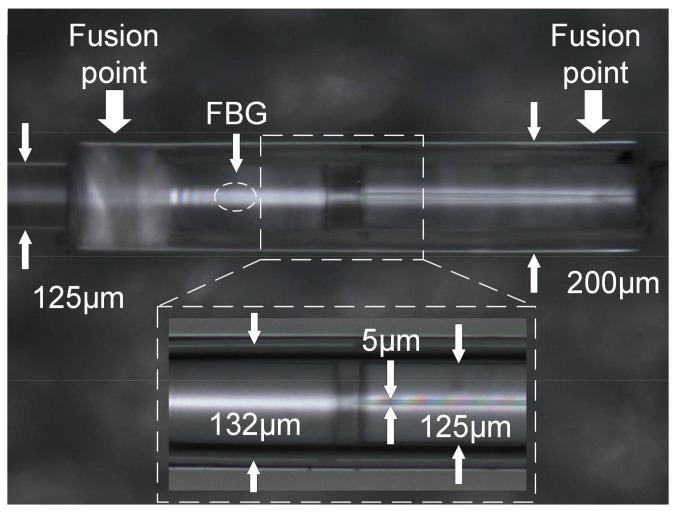
Microscopic image of the fiber-optic FP gas pressure sensor.

A microscopic image of the sensor is shown in Figure 3. The FBG peak reflectivity, bandwidth and center wavelength are 65%, 0.5 and 1575 nm, respectively. The distance between the end face of the SMF and the HST, and the inner diameter of the HST have a great influence on the spectrum. The contrast of the interference spectrum decreases as the inner diameter increases. After comprehensive consideration, we chose the HST with an inner diameter of 5 μm. The specifications of the SMF, silica casing and HST of the sensor are listed in Table 1. Due to the protection of the silica casing, the two reflection surfaces of S_1_ and S_2_ were kept in parallel. The fusion points are at both ends of the casing, which can better protect the two reflection surfaces from being damaged. At a certain temperature, the silica casing prevents the HST from collapsing due to overheating. By controlling the insertion length of the HST, the length of the FP cavity can be flexibly controlled.

**Figure 4:**
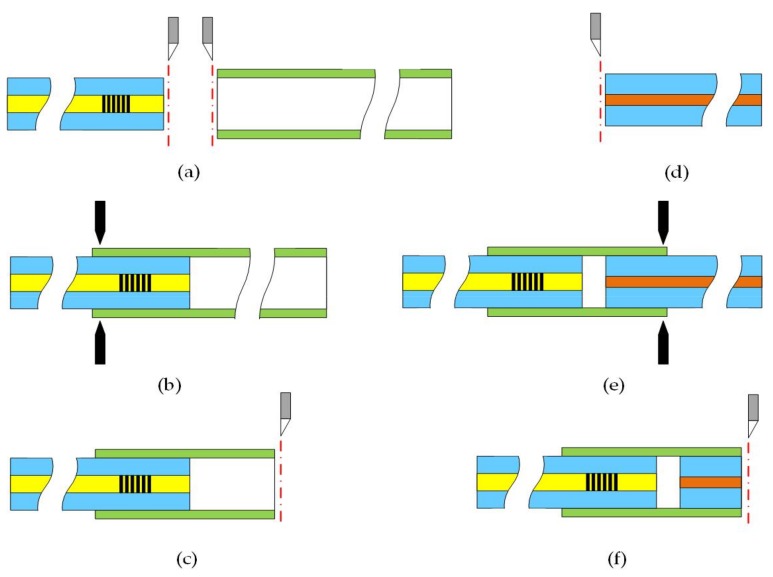
The schematic of the fabrication process. (**a**) Well-cleaved the SMF and silica casing. (**b**) Fusing the SMF and silica casing together at the end of the silica casing. (**c**) Cleaved the silica casing. (**d**) Well-cleaved the HST. (**e**) Fusing the silica casing and HST together at the other end of the silica casing. (**f**) Cleaved the excess of the HST.

Figure 4 illustrates the fabrication process of the sensor. The sensor was fabricated as follows. Firstly, the SMF and the silica casing well were cleaved with a fiber cleaver, as shown in Figure 4a. Inserting the SMF into the silica casing by the manual fusion procedure of the splicer (FITEL, S183 version 2, Tokyo, Japan). Discharged 1–2 times at the left fusion point, as shown in Figure 4b. The parameters of the splicer were set as A. The silica casing was cleaved at a suitable distance from the end of the SMF with the fiber cleaver under a microscope, as shown in Figure 4c. Next, cleaved the HST well with the fiber cleaver and inserted the HST from the opposite end of the silica casing by the manual fusion procedure of the splicer, as shown in Figure 4d. Kept a suitable distance with the end of the SMF to form the FP cavity. Discharged 1–2 times at the right fusion point, as shown in Figure 4e. The parameters of the splicer were set as B. Finally, the excess of the HST was cleaved off under a microscope, as shown in Figure 4f. The fusion parameters are shown in the Table 2.

**Figure 5:**
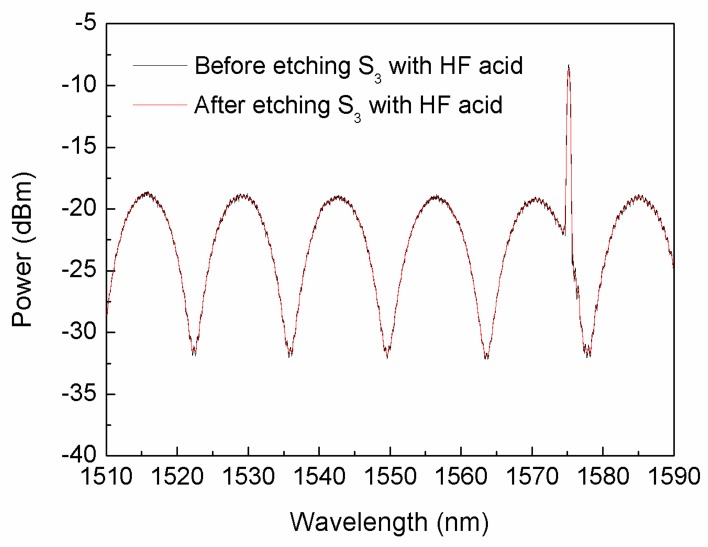
Interference spectrum of the fiber-optic FP gas pressure sensor with FP interferometer and FBG.

The incident light reflected by the FBG, S_1_, S_2_ and S_3_ form an interference spectrum, which is shown in black in Figure 5. From the result, in order to get the interference spectrum with less influence, etched the S_3_ with hydrofluoric acid, the interference spectrum is shown in red in Figure 5. The wave superimposed on the interference spectrum is due to the tiny distance between the S_1_ and the FBG when cleaved under the microscope. The results well agreed with the simulation as shown in Figure 2.

## 4. Experiments and Results

**Figure 6:**
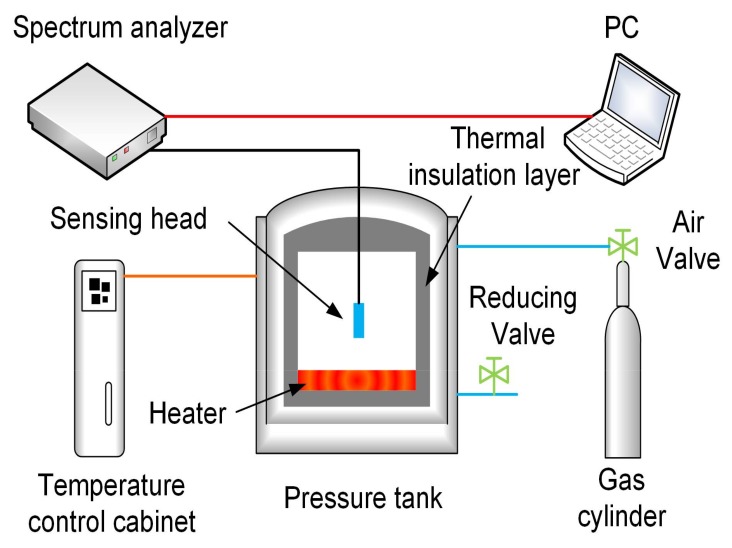
Experimental setup for pressure and high-temperature test.

The experimental test system was set up with computer, spectrum analyzer, pressure tank, temperature control cabinet and gas cylinder, as shown in Figure 6. The light beam propagates from the spectrum analyzer (SM125, Micron Optics Inc., Atlanta, GA, USA), and it transmits into the sensor proposed in this paper for pressure and temperature detection. The sensor was placed into the pressure tank for the pressure tests at different stable temperatures. The temperature produced by the heater was controlled with the temperature control cabinet, and the pressure was adjusted by the gas cylinder. The temperature and pressure in the tank were measured by a calibrated temperature sensor and a calibrated pressure sensor which the accuracy were ±0.1% F.S and ±5‰ MPa respectively. The data recorded by the spectrum analyzer with a resolution of 1 pm under the range of 1510 nm to 1590 nm is processed with the PC.

**Figure 7:**
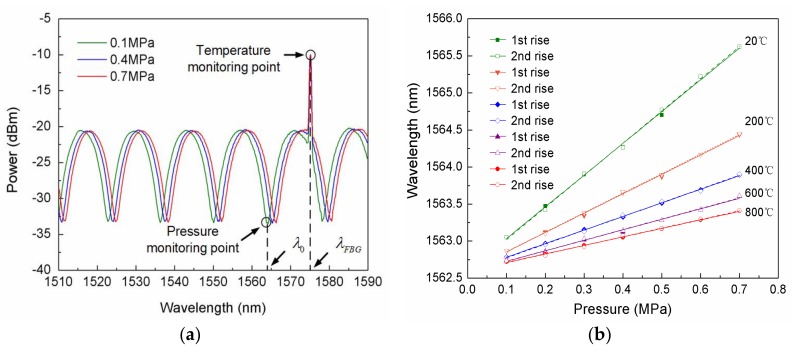
Interference spectrum: (**a**) FP interferometer with stable FBG; (**b**) The shifts of wavelength with pressure at 20, 200, 400, 600 and 800 °C.

The calibration process of the sensor in the nitrogen environment is as follows. Put the sensor in the tank and get as close to the heater as possible to detect the operating temperature, the pressure in the tank was changed by filling the tank with the pure nitrogen. Adjust the pressure in the tank from approximately 0.1 MPa (barometric pressure) to 0.7 MPa at 20, 200, 400, 600 and 800 °C. At each 0.1 Mpa, recorded the interference spectrum after maintaining the temperature for 5 min. The experimental results show that the interference spectrum shifts to a long wavelength consistently as the pressure increases at 20, 200, 400, 600 and 800 °C. Figure 6a shows the spectrum shift at the pressure of 0.1, 0.4 and 0.7 MPa under 20 °C. Figure 7a is the shift of the spectrum when the pressure stabilized at 0.1, 0.4 and 0.7 MPa at 20 °C. From Figure 7a, it can be seen that the sensor has a sensitive response to the pressure. Due to the temperature being maintained at 20 °C, the center wavelength of the FBG has no shift. The pressure monitoring point and temperature monitoring point are shown in Figure 6a. The initial FPI “peak” and center wavelength of the FBG are $\lambda_{0}=1563.0524$ and $\lambda_{FBG}=1574.8658\;\mathrm{nm},$ respectively. The fitting curves of the wavelength at different pressures under 20, 200, 400, 600 and 800 °C are shown in Figure 7b. From Figure 7b, it can be seen that the wavelength has a good linear relationship with the pressure, and duplicate experimental results are in a good agreement. This proves that the sensor has a good linearity and repeatability at different temperatures. The sensitivities at 20, 200, 400, 600 and 800 °C are 4.28, 2.62, 1.83, 1.41 and 1.15 nm/MPa, respectively.

**Figure 8:**
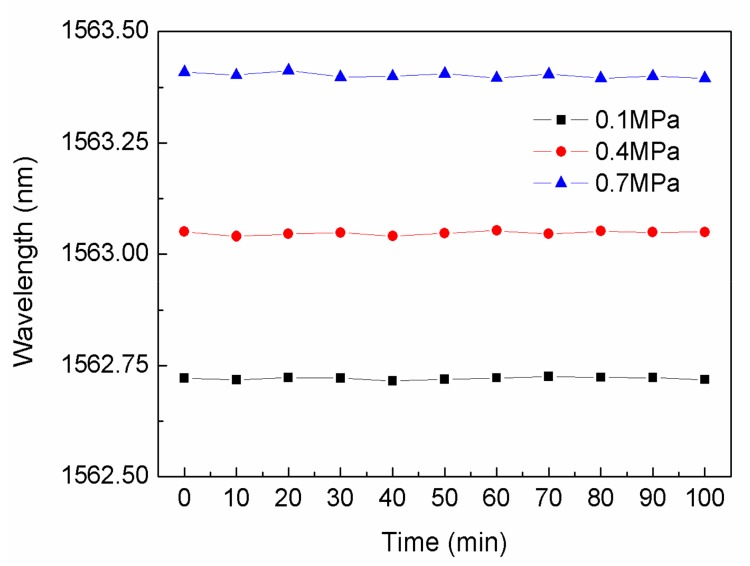
The stability test results of the proposed sensor at 0.1, 0.4 and 0.7 MPa under 800 °C for 100 min.

The stability of the sensor proposed in the paper was measured under pressure at 0.1, 0.4 and 0.7 MPa at 800 °C for about 100 min, the results is shown in Figure 8, where it can be seen that the sensor proposed in the paper had good stability with slight variations in the wavelength response for at least 100 min.

**Figure 9:**
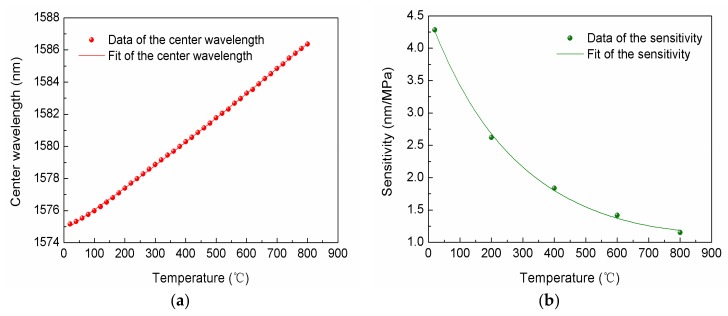
Fitting curves of the relationship of: (**a**) FBG center wavelength and temperature; (**b**) sensitivity and temperature.

The FBG used in the sensor has been annealed and reached a stabilization point. The relationship between the FBG center wavelength and temperature is shown in Figure 9a. It can be seen that the FBG center wavelength has a good linear relationship with the temperature and shifts to a long wavelength as the temperature rises from 20 to 800 °C. The FBG center wavelength can be fitted with the relationship of the Equation (5) with A = 1574.7756, B = 0.0125, C = 3.8889 × 10^−6^ and D = −1.6458 × 10^−9^, where $\lambda_{FBG}$ is the FBG central wavelength. The fitting degree is 99.98%. From Figure 7b, the relationship between the wavelength and pressure at 20, 200, 400, 600 and 800 °C can be obtained by the wavelength at 0.1 MPa and the sensitivity at the corresponding temperature. The wavelength of pressure monitoring point at 20, 200, 400, 600 and 800 °C under 0 MPa, calculated by the relationship between the wavelength and pressure, are 1562.6064, 1562.5912, 1562.5987, 1562.5856 and 1562.5966 nm, respectively. The wavelength has almost no drift proving the temperature compensation structure is effective. The average of the wavelength is 1562.5957 nm. From Figure 7b, we can fit the wavelength values corresponding to the pressure monitoring point at each step from 0.1 Mpa to 0.7 Mpa to get the sensitivities of the sensor at 20, 200, 400, 600 and 800 °C. The sensitivities of the sensor at different temperatures are fitted with the relationship of Equation (6) with a = 1.5087, b = −0.0029 and c = 1.5703 × 10^−6^. The fitting of the sensitivities is shown in Figure 9b. In Figure 9b, it can be seen that the sensitivities have an exponential relationship with the temperature and the fitting degree is 99.74%.

(5) $$\lambda_{FBG}=A+B\cdot T+C\cdot T^{2}+D\cdot T^{3}$$

(6) $$y=e^{a+b\cdot T+c\cdot T^{2}},$$

**Figure 10:**
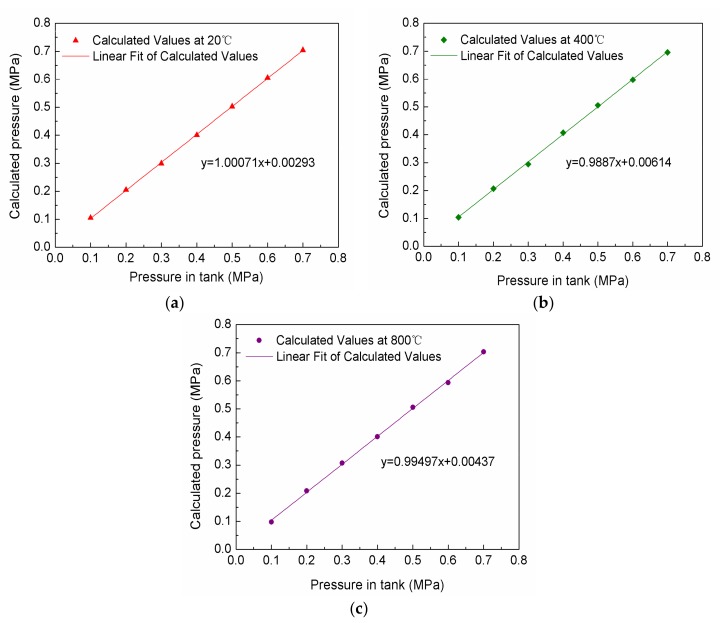
Temperature decoupling results: (**a**) at 20 °C; (**b**) at 400 °C; (**c**) at 800 °C.

By using the temperature decoupling method, the calculated values and the fitting curves of the calculated values at 20, 400 and 800 °C are respectively shown in Figure 10a–c. In Figure 10, the pressures measured by the pressure gauge of the high-temperature pressure compounding platform are the actual values. From Figure 10, it can be seen that the calculated values are in good agreement with the actual values, and there is only a slight error between the calculated values and the actual values. The slopes of the fitting curves of the calculated values at 20, 400 and 800 °C are 1.00071, 0.9887 and 0.99497, respectively, which are very close to 1. This proves that the presented sensor is quite reliable. The maximum error of the calculated values at 20, 400 and 800 °C are 5.3, 7.6 and 8.1 KPa respectively. The non-linearity errors of the calculated values are 0.88%, 1.28% and 1.34%, respectively, which is less than that of the conventional gas pressure sensors on the market. The reason why the deviation of the calculated values from the actual value increases with the temperature increases may be due to the deviation of the sensitivity fitting increases as the temperature increases. In the future work, we will conduct a better study of the fitting method. We believe it will be better to improve the accuracy of the temperature decoupling method.

Because the pressure measurement results are related to the refractive index of the gas, the sensor can be used for single-component gas or mixed-component invariant gas detection applications, such as, pressure monitoring of high-temperature steam in the pipelines of a thermal power station, and pressure measurements under laboratory conditions. In summary, the sensor can be used in the monitoring the single-component or mixed-component invariant gas environments by calibrating the sensor according to the environment used. If the gas mixture composition changes during the measurement, the sensor would not suitable for such an environment, unless we realize the situation after the change.

## 5. Conclusions

To summarize, a diaphragm-free fiber-optic gas pressure sensor for high temperature environments based on pressure changes with gas density, which provides a method of pressure measurement at high temperature is presented. It uses a FBG to realize the in-situ temperature measurement. The pressure sensitivity of the fiber-optic FP gas pressure sensor is 4.28 nm/MPa with a high linear pressure response over the range of 0.1–0.7 MPa and the temperature sensitivity is 14.8 pm/°C within the 20–800 °C range. The sensor has less than 1.5% non-linearity at different temperatures by using a temperature decoupling method. The sensor has advantages of extremely low temperature coefficient, compact structure, small size, simple production and low cost which will help the sensor offer the excellent features required for pressure measurements in high temperature applications.

## Figures and Tables

**Table 1:** The specs of parts used in the sensor.

Part Name	Type	Inner Diameters (um)	Outer Diameters (um)
SMF	G652D, Yangtze Optical Fiber and Cable Co., Ltd., Wuhan, China	9	125
Silica casing	YN132200, Yongnian Ruipu Chromatogram Equipment Co., Ltd., Hebei, China	132	200
HST	YN005125, Yongnian Ruipu Chromatogram Equipment Co., Ltd.	5	125

**Table 2:** Fusion parameters.

	Clean Intensity	Clean Time	Fusion Intensity	Fusion Time
A	200 units	250 ms	100 units	600 ms
B	120 units	180 ms	100 units	800 ms
